# TAVI: Simplification Is the Ultimate Sophistication

**DOI:** 10.3389/fcvm.2018.00096

**Published:** 2018-07-18

**Authors:** Mariama Akodad, Thierry Lefèvre

**Affiliations:** ^1^Ramsay Générale de Santé, Institut Cardiovasculaire Paris Sud, Massy, France; ^2^Centre Hospitalier Universitaire Arnaud de Villeneuve, Montpellier, France

**Keywords:** TAVI, simplification, minimalist, vascular complications, temporary pace maker, hospital stay duration

## Abstract

Since its introduction in 2002, TAVI has evolved dramatically and is now standard of care for intermediate risk patients when the femoral approach can be implemented safely. The development of innovative transcatheter heart valves (THVs) and refinement of technical skills have contributed to the decrease in complication rates associated with TAVI^4^. Increased experience, smaller sheaths, rigorous pre-procedural planning and improved vascular closing techniques have resulted in markedly lower rates of vascular complications. The next step is the simplification of the procedure, which should contribute to a further decrease in complications, and also reduce procedural time, hospital stay as well as staff workload and costs. Moving to conscious sedation, no predilatation, no temporary pace maker and use of the radial approach as the contralateral approach are all instrumental in achieving this ultimate refinement.

## Introduction

Since the first successful procedure was carried out in 2002 (1), transcatheter aortic valve implantation (TAVI) has gradually been established as an alternative to conventional surgery in patients with severe aortic stenosis contra-indicated to surgery or at high surgical risk (2). In 2017, during the last ESC meeting, TAVI indications were extended to intermediate risk patients when the transfemoral approach (TFA) is feasible (3).

Improvements in technique, devices, operator's experience, and patient selection have contributed to a dramatic decrease in procedural complications, thus allowing further technical simplification at every step of the procedure (4–9). In this paper, our aim is to describe how to simplify the technique at each stage of the procedure in order to turn it into a “PCI-like” procedure and to discuss how this may improve TAVI outcomes.

## Pre-procedural evaluation and procedural setting

Patient clinical and anatomical criteria may influence per- and post- procedural outcomes. Therefore, a truly minimalist approach should be considered only when femoral access is possible. Recently, Barbalios et al. compared minimalist TAVI performed in the catheterization laboratory to standard TAVI performed in the hybrid room demonstrating shorter procedure and intensive care unit time, as well as reduced hospitalization duration and costs in the minimalist approach group without differences in terms of short- and long-term survival (10).

Multislice CT (MSCT) is instrumental in procedural simplification. Image quality and optimal analysis are therefore crucial for anticipating the potential difficulty of the procedure as well as for optimal valve selection and working view. The role of MSCT has also been central in allowing a shift from general anesthesia to conscious sedation by obviating the need for transesophageal echocardiography (TOE) during the procedure. MSCT became the gold standard for evaluation of the aortic root in our center in 2009.

The use of the TRA for preprocedural evaluation of the coronary arteries is also part of the simplification process. It helps not only to reduce the risk of vascular complications related to the screening phase, but also to assess femoral access by performing a selective bilateral iliac injection using a long multipurpose catheter. Recently, screening of coronary artery disease and ad hoc percutaneous coronary intervention (PCI) during TAVI has been described by Barbanti et al. showing to be feasible without increased periprocedural complications (11).

**Figure 1:**
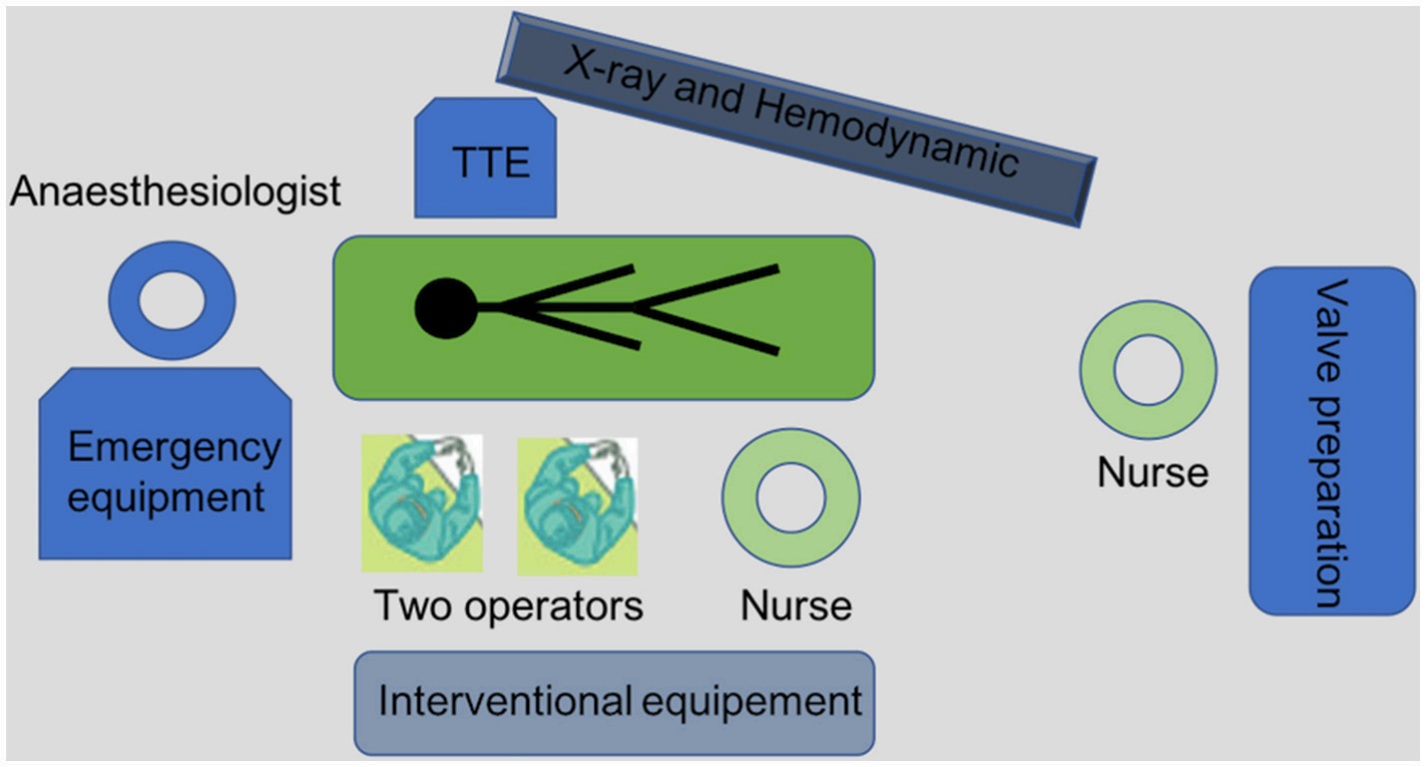
Equipment and procedural set-up for a “minimalist” TAVI procedure.

A minimalist approach can be performed in routine practice with two operators, two nurses and an anesthesiologist (Figure 1). A cardiac surgeon and an echocardiographist are not mandatory in the room but should be available.

## From general anesthesia to conscious sedation

Although the first in-man TAVI cases in Rouen were initially performed on conscious sedation (1), the procedure was commonly carried out under general anesthesia between 2002 and 2008 in Europe. It remains standard practice in the majority of cases in North America (12). However, it was Alain Cribier's idea that TAVI should be a “PCI- like” procedure. Potential advantages of general anesthesia are patient's procedural comfort, possibility of using TOE and rapid conversion to surgery when complications occur (13–15). Conversely, many issues are related to general anesthesia such as hemodynamic instability, higher need for inotropic drugs, higher risk of bleeding, increased risk of pulmonary infection, extubation difficulty or delay in patients with chronic pulmonary disease, late complication identification such as stroke or aortic complications and finally, longer procedural duration, hospital stay, higher staff workload, and global costs (16–18). In the France 2 and the France TAVI registries, the adoption of local anesthesia with conscious sedation has progressively increased from 30% in 2010 to 70% in 2017 (15, 19). In a recent meta-analysis, outcomes of both approaches were similar with respect to in-hospital mortality, conversion to open-heart surgery, major vascular complications, acute kidney failure and stroke (17). Cross-over to general anesthesia was observed in only 6%. Conversely, catecholamine requirement and transfusion were less frequent in patients on conscious sedation, and duration of intensive care unit and global hospital stay was also shorter (17, 20, 21). No difference concerning neurocognitive outcomes was highlighted between both approaches (21).

Data from registries demonstrated the feasibility, safety, and cost-effectiveness of local anesthesia with conscious sedation in comparison to general anesthesia, with potential advantages in terms of bleeding and hospitalization length. It has been adopted as the default approach in our center since April 2009.

## From surgical cut down to percutaneous access

**Figure 2:**
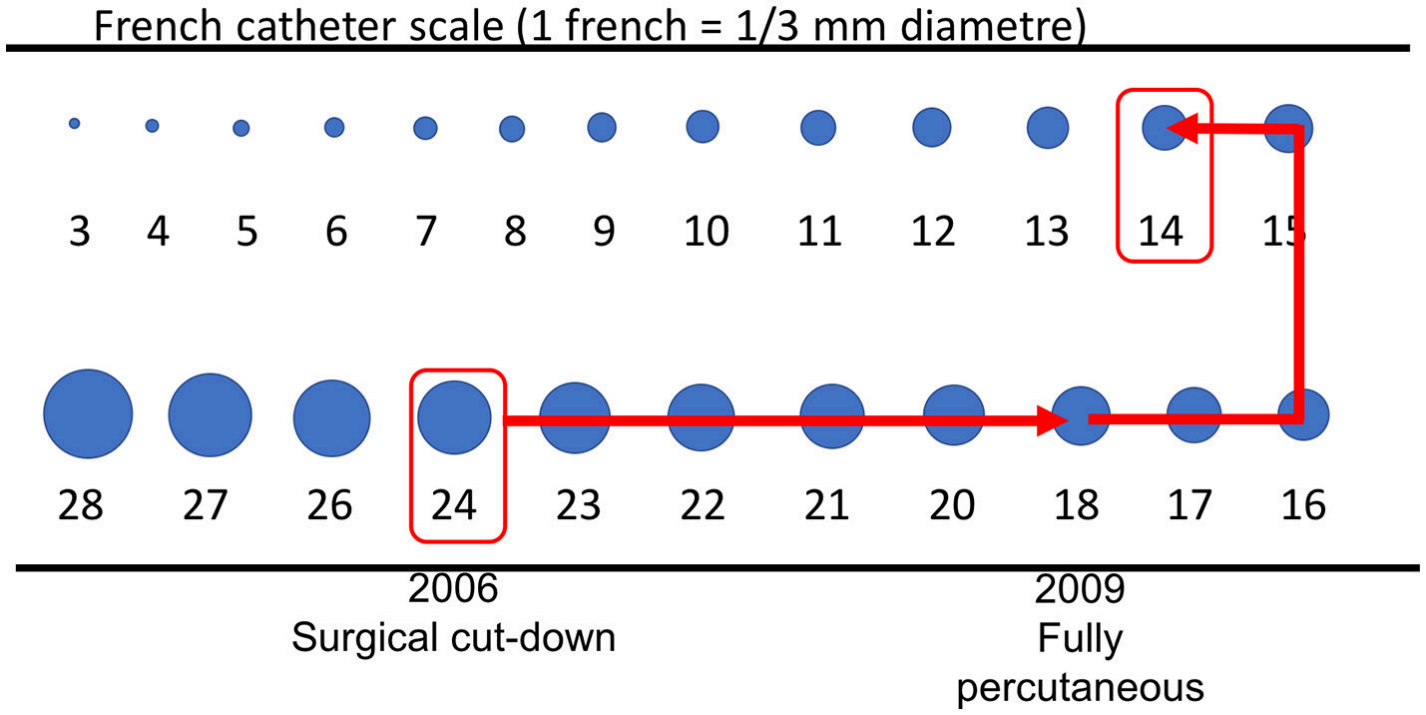
Sheath size from first to last generation devices.

Initially, TAVI procedures were performed exclusively via surgical cut down (1). Over the past decade, sheath diameter has been gradually reduced to 14–16 French with the last generation percutaneous heart valves (Figure 2). TFA is currently the default access route, with superior outcomes than transapical route and other transvascular approaches as carotid, aortic, axillary, and caval-aortic. Alternative transvascular routes may be considered anly in case of unsuitable femoral access (22).

Percutaneous closure has been progressively adopted in routine practice in most centers for TF TAVI procedures (23). Indeed, even though the surgical approach has been reported to be associated with a low rate of vascular complications and to provide a more direct control of haemostasis (24), percutaneous closure is a less invasive technique and may result in shorter hospital stay (25). With the refinement of the TAVI procedure, better patient preprocedural screening, increased operator experience, and device improvement, the percutaneous approach has become more simple, less time consuming, thus allowing a reduction in staff workload. The Prostar technique was introduced in our center in 2009 and we moved progressively to Proglide (Abbott Vascular Devices, Redwood City, CA, United States) preclosing in 2015-2016 (26) because this technique was simpler and less costly. In 2016, a lower risk of vascular complications was reported with the use of 2 Proglide devices in comparison with Prostar (27). New collagen-based closure devices were recently described in TAVI procedure as the MANTA closure device with similar results than suture based closure devices (28).

In addition to the selection of the most appropriate percutaneous device, the percutaneous technique should be rigorous in order to limit access site complications. Indeed, the common femoral artery puncture site should be carefully selected on the CT-scan or angiography before the procedure. During the procedure, puncture should be performed under angiographic or ultra-sound guidance at the center of the anterior arterial wall (29). Percutaneous closure devices should be subsequently deployed as previously described (30).

Thus, percutaneous transfemoral access is as safe as the surgical approach and feasible in the majority of cases with a very high rate of success after the learning phase. Most vascular complications can be managed percutaneously. It is an essential component of TAVI's simplification process allowing early discharge.

## From contralateral femoral access to radial access

Although vascular complications dramatically decreased in parallel with enhanced operator experience, availability of low profile sheaths and better patient selection, 25–30% of these complications occurred at the contralateral femoral access site, (9). Therefore, using the TRA as a secondary access appears to be very promising (29–31). We have been using this approach since 2016 and have observed a 50% reduction in vascular complications. The radial artery (right or left) is punctured and a 40 cm 6 Fr hydrophilic sheath with a side port for blood pressure measurement is subsequently inserted through the radial artery. A 125 4 or 5 Fr multipurpose (MP) catheter is advanced over a standard 0.35 guide wire to the common iliac artery in order to obtain a reference image and guide the puncture. After the puncture, the MP catheter is retrieved and a pig-tail is advanced in the ascending aorta via the 0.35 guide wire to perform aortography before and after TAVI. At the end of the procedure, prior to access closure, the MP catheter is re-advanced to the common femoral artery in order to check the final result of the closure (29). In cases of vascular complication, a long 120 cm 5 Fr catheter (Optimed, Germany) can be positioned in the common femoral artery to perform femoral artery balloon inflation or stent implantation. In rare cases where a covered stent is needed, a larger balloon can be used through the Optimed catheter in order to close temporarily the iliac or femoral artery, while a cross-over femoral approach is implemented (29). Indeed, in our practice, even if the radial secondary access is our default approach, the controlateral femoral access should be available immediately in case of failure or emergent need of cross-over.

Therefore, by reducing contralateral vascular complications and simplifying the procedure, TRA will probably follow the predominant tendency observed in other interventional cardiology settings and become the gold standard contralateral approach for TAVI.

## From venous stimulation to LV guide wire pacing

**Figure 3:**
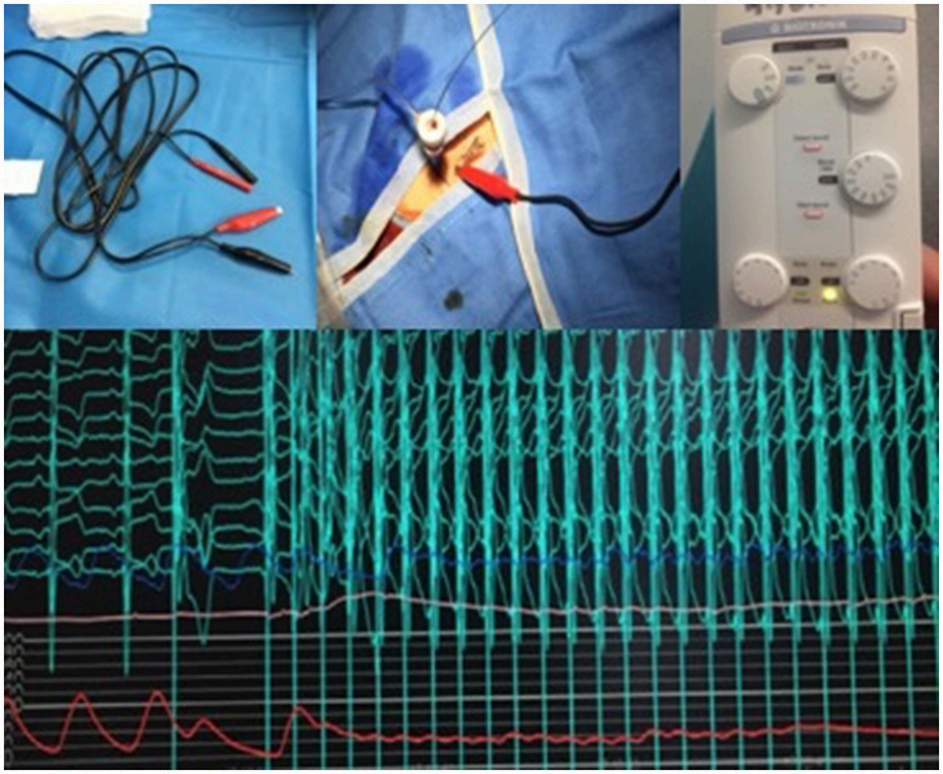
From left to right and up to down: Alligator clips with negative clip (black) and positive clip (red). After insertion of Sheath, a 22G needle is inserted subcutaneously through the skin, close to the femoral sheath and the positive clip is connected to the needle while the negative clip is connected to the guidewire. Setting of the temporary pacemaker with maximal output and minimal sensitivity. Pacing efficacy at 180 beats per minute with the LV wire and drop in blood pressure.

During balloon valvuloplasty (BAV) or balloon expandable TAVI procedures, rapid ventricular pacing is mandatory. Traditionally, rapid pacing is performed through a venous access with temporary pacemaker implantation (1). However, this technique may be challenging in anatomic variations and may lead to increased X-ray exposure and complications (32) such as hematoma, arterio-venous fistula, thrombosis or right ventricle perforation. Recently, rapid ventricular pacing through the left ventricle guide wire has been described as a way of simplifying the procedure by eliminating the need for additional vascular access during TAVI (33). This approach was adopted in 2016 in our center. Briefly, a 22 G needle is inserted subcutaneously near the femoral sheath. Alligator clips are then connected to the left ventricle guide wire (negative clip) and to the needle (positive clip) following insertion of the delivery system close to the aortic valve. The rapid pacing is then tested using maximal output and minimal sensitivity (Figure 3). Valve implantation is then carried out under rapid pacing. In the presence of high-degree conduction disturbance, stimulation can be performed with this technique while a temporary pacemaker is inserted through a venous access, more frequently through brachial vein access to limit femoral vascular complications.

This new technique has been shown to be feasible and safe, allowing stable stimulation with a low rate of complications and a potential reduction in procedural time. A randomized trial comparing left ventricle guide wire rapid pacing to conventional pacing (Easy TAVI) is ongoing in France (NCT02781896).

## Valve implantation without predilation

In the early days of TAVI, BAV was considered a mandatory step. However BAV have been shown to be associated with a higher risk of cerebral embolization, and severe acute aortic regurgitation may occur after predilatation in up to 3% of cases. TAVI without BAV was evaluated for the first time in 2011 by Grübe et al. (34) and was shown to be feasible in non-randomized studies (35, 36). Currently, improvements in new generation devices including paravalvular skirts, the ability of repositioning of the valve, lower profile of delivery system, and of the prosthesis provide more favorable outcomes (22). Therefore, BAV seems no longer essential during TAVI procedures, and consequently, TAVI without predilation is routinely implemented in many centers.

We moved progressively to this approach between 2012 and 2015 in our center. Today, more than 90% of cases are performed without predilatation. Only very complex anatomies or highly calcified valves are predilated before valve deployment (5–10%). Post dilatation is performed mainly after self-expandable valve deployment in the presence of significant paravalvular leak or transvalvular gradient (10–15%).

Thus, avoiding balloon predilatation may reduce complication rates, decrease the need for permanent pace-maker and reduce procedural time. A large randomized trial (37) with the Sapien 3 valve is on-going in France (NCT02729519).

## Post-procedure management:

### From intensive care unit (ICU) to conventional cardiology unit (CCU)

After TAVI, systematic close monitoring with special attention to hemodynamic and cardiac rhythm is mandatory to allow early detection of periprocedural complications. In many centers, monitoring is performed for at least 12 to 24 h in the ICU before transferring the patient to a CCU after clinical and paraclinical status re-assessment. Recently, TAVI without subsequent ICU admission has been evaluated and has been shown to be feasible and safe in selected patients after rigorous preprocedural and postprocedural evaluation (38). Indeed, this new strategy adopted in our center in 2017 may obviate ICU admission in up to one third of cases and should be considered a part of the “minimalist” approach.

### Short hospitalization

Early discharge was evaluated in the literature demonstrating safety in patients with hospitalization duration shorter than 48 h (39). Indeed, the median length of hospitalization was 1 day in the early discharge group with no differences between early discharge and discharge after 48 h in terms of 1-month mortality, stroke and readmission. A “minimalistic” TAVI procedure with local anesthesia, no predilatation, urinary catheter avoidance and early removal of temporary pacemaker was predictive of early discharge in this study. Current outcomes of early discharge after TAVI are summarized in Table 1. Shortening hospital stay is also an essential component of the TAVI simplification process with a potential reduction in procedural costs and need for rehabilitation but may be studied in large studies to ensure safety without increased risk of outcomes or readmission.

**Table 1:** Current outcomes of early discharge after TAVI.

**Study**	**Patients**	**Early discharge, n (%)**	**Timing of early discharge**	**30-days mortality, n (%)**	**Rehospitalization within 30-days, n (%)**
Durand et al. 40	337	121 (36)	Within 3 days	0 (0)	4 (3.3)
Noad et al. 41	120	26 (21.7)	Same/next day	0 (0)	1 (3.84)
Serletis-Bizios et al. 42	130	76 (59)	Within 3 days	1 (1.3)	3 (3.94)
Lauck et al. 43	393	150 (38.2)	Within 2 days	1 (0.7)	12 (8)

## Conclusion

TAVI simplification has already been adopted in routine practice in experienced centers, resulting in a low rate of complications, shorter procedural time, improved patient comfort, as well as decreased costs and staff workload. However, rigorous patient selection, and risk stratification are key factors in ensuring successful “PCI-like” procedures. On-going randomized trials may confirm preliminary results, thus leading to a “simple” but not “simpler” procedure in the near future with lower profile devices.

## Author contributions

All authors listed have made a substantial, direct and intellectual contribution to the work, and approved it for publication.

### Conflict of interest statement

TL is proctor for edwards and Abbott vascular MA received research grants from Edwards Lifescience and Medtronic.

## Abbreviations

BAV: balloon aortic valvuloplasty
CCU: conventional cardiology unit
ICU: intensive care unit
LV: left ventricle
MP: multipurpose
MSCT: multislice CT
PCI: percutaneous coronary intervention
TAVI: transcatheter aortic valve implantation
TFA: transfemoral approach
TOE: transesophagal echocardiography
TRA: transradial approach.
